# Expression and Purification of the Human Voltage-Gated Proton Channel (hH_v_1)

**DOI:** 10.21769/BioProtoc.5344

**Published:** 2025-06-20

**Authors:** Emerson M. Carmona, D. Marien Cortes, Luis G. Cuello

**Affiliations:** 1 Cell Physiology and Molecular Biophysics Department and the Center for Membrane Protein Research. Texas Tech University Health Sciences Center, Lubbock, TX, USA; 2 Department of Neurobiology and Biophysics, University of Washington, Seattle, WA, USA

**Keywords:** Voltage-gated proton channel (H_v_1), Membrane protein purification, Bacterial overexpression, Autoinduction, Anzergent 3–12 solubilization, IMAC purification

## Abstract

The voltage-gated proton channel (H_v_1) is a membrane protein that dissipates acute cell proton accumulations. To understand the molecular mechanisms explaining H_v_1 function, methods for purifying the protein are needed. Previously, methods were developed for expressing and purifying human H_v_1 (hH_v_1) in yeast and later in bacteria. However, these methodologies produced low protein yields and had high production costs, considerably limiting their usefulness. The protocol described in this work was developed to overcome those limitations. hH_v_1 is overexpressed in bacteria, solubilized with the detergent Anzergent 3–12, and purified by immobilized metal affinity chromatography (IMAC) and size-exclusion chromatography (SEC). Our protocol produced higher protein yields at lower costs than previously published methodologies.

Key features

• hH_v_1, containing a poly-His tag followed by an enterokinase cutting site in its N-terminus, is overexpressed in *E. coli* by autoinduction.

• The detergent Anzergent 3–12 is used to solubilize and purify hH_v_1 using nickel-immobilized metal affinity chromatography (IMAC).

• The entire procedure can be performed in 6 days.

## Graphical overview


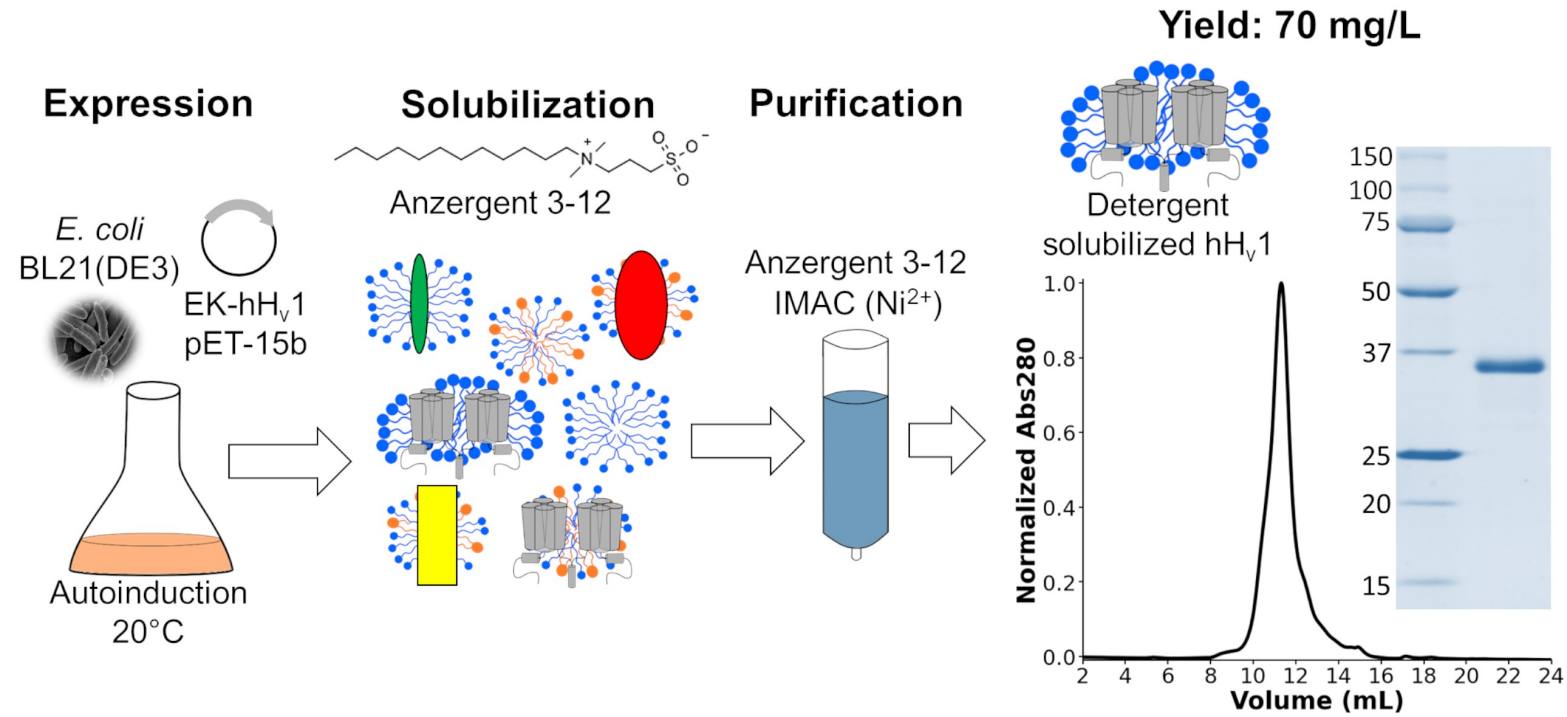


## Background

The voltage-gated proton channel (H_v_1) is a membrane protein that contains a highly selective permeation pathway for protons [1,2]. The opening of this proton permeation pathway is regulated by voltage [3,4], pH gradients [4,5], mechanical forces [6], and ligand binding [6–9]. The structural basis of these H_v_1 biophysical properties is still poorly understood, including its permeation pathway location, sensitivity to pH gradients, mechanosensitivity, cooperativity of opening, and ligand binding sites. This missing knowledge is needed to use H_v_1 as a therapeutic target for diseases such as immune disorders [10], diabetes [11], inflammatory pain [12], and cancer [13]. Most biophysical and structural studies require high quantities of stable and functional recombinant H_v_1, which has been challenging to achieve. Initially, insect cells were used to express a chimeric recombinant mouse H_v_1 to obtain its structure by X-ray crystallography [14]; later, the human H_v_1 (hH_v_1) was expressed and purified using yeasts [15]. Finally, a new methodology was developed to produce hH_v_1 in *E. coli* for an electron paramagnetic resonance (EPR) study [16]. Other authors have used this last protocol to study hH_v_1 using nuclear magnetic resonance (NMR) [17] and single-molecule Förster energy transfer (sm-FRET) [18]. Nevertheless, this protocol had two main limitations: i) the protein yield was low (0.7 mg per liter of culture), and ii) the expensive detergents Fos-choline 12 and 14 were used to purify the protein. Recognizing these limitations, we optimized each step of the protocol to produce a new method that increased the protein yield up to 70 mg per liter of culture and decreased costs using the more economical detergent Anzergent 3–12. The final purified hH_v_1 protein is stable and functional [19]. Here, we describe such a protocol in detail to accelerate hH_v_1 research.

## Materials and reagents

**Biological materials**

1. BL21-Gold(DE3) competent cells (Agilent, catalog number: 200131)

2. pHis-EK-hH_v_1.pET15-b (source and sequence in Dataset S1)

3. Mouse α-penta-His antibody (Qiagen, catalog number: 34660)

4. StarBright Blue 700 goat α-mouse IgG (Bio-Rad, catalog number: 12004158)

**Reagents**

1. Tryptone (Fisher, catalog number: BP1421)

2. Yeast extract (Fisher, catalog number: BP1422)

3. NaCl (Sigma-Aldrich, catalog number: S9888)

4. PEG (Sigma-Aldrich, catalog number: P3640)

5. MgSO_4_·7H_2_O (Sigma-Aldrich, catalog number: M-9397)

6. MgCl_2_·6H_2_O (Fisher, catalog number: BP214)

7. Glycerol (Fisher, catalog number: G33-4)

8. Sterile-filtered DMSO (GoldBio, catalog number: D-361)

9. Ampicillin (sodium) (GoldBio, catalog number: A-301)

10. KCl (Fisher, catalog number: P217)

11. Glucose (Sigma-Aldrich, catalog number: G6152)

12. 1,000× trace metal mixture (Teknova, catalog number: T1001)

13. Na_2_HPO_4_·7H_2_O (Sigma-Aldrich, catalog number: S9390)

14. KH_2_PO_4_ (Sigma-Aldrich, catalog number: P5655)

15. NH_4_Cl (Sigma-Aldrich, catalog number: 213330)

16. Na_2_SO_4_ (Sigma-Aldrich, catalog number: 239313)

17. Glycerol (Fisher, catalog number: G33-4)

18. α-lactose monohydrate (Sigma-Aldrich, catalog number: L2643)

19. Tris buffer (Invitrogen, catalog number: 15504020)

20. Sodium dodecyl sulfate (SDS) (Bio-Rad, catalog number: 1610302)

21. Bromophenol blue sodium salt (Sigma-Aldrich, catalog number: B7021)

22. 2-Mercaptoethanol (Sigma-Aldrich, catalog number: M3148)

23. 10% v/v antifoam 204 (Teknova, catalog number: A6427)

24. Phenylmethanesulfonyl fluoride (PMSF) (Sigma-Aldrich, catalog number: P7626)

25. Isopropanol (Fisher, catalog number: A416)

26. Benzamidine hydrochloride hydrate (Sigma-Aldrich, catalog number: B6506)

27. Lysozyme egg white (GoldBio, catalog number: L-040)

28. DNase I, bovine pancreas (GoldBio, catalog number: D-300)

29. 10× Tris/glycine/SDS running buffer (Bio-Rad, catalog number: 1610772)

30. 12% Mini-PROTEAN^®^ stain-free^TM^ protein gels (Bio-Rad, catalog number: 4568045)

31. Precision plus protein unstained standards (Bio-Rad, catalog number: 1610363)

32. Precision plus protein dual color standards (Bio-Rad, catalog number: 1610374)

33. Ethanol (Fisher, catalog number: BP28184)

34. 5× Transfer buffer (Bio-Rad, catalog number: 10026938)

35. KCl (Fisher, catalog number: P217)

36. Tween 20 (Bio-Rad, catalog number: 1706531)

37. Low-fat dry milk (Milkman)

38. TCEP HCl (GoldBio, catalog number: TCEP)

39. Anzergent 3–12 (Anatrace, catalog number: AZ312)

40. Imidazole (Thermo Scientific Chemicals, catalog number: 396745000)

41. HCl (Fisher, catalog number: AC423795000)

**Solutions**

1. Luria-Bertani broth (Miller) (LB) (see Recipes)

2. 1 M MgSO_4_ (see Recipes)

3. 1 M MgCl_2_ (see Recipes)

4. 50% glycerol (see Recipes)

5. Transforming and Storage Solution (TSS) (see Recipes)

6. 100 mg/mL ampicillin (see Recipes)

7. 3 M KCl (see Recipes)

8. 4 M NaCl (see Recipes)

9. 40% (w/v) glucose (see Recipes)

10. Super optimal broth with catabolite repression (SOC) (see Recipes)

11. Terrific broth (see Recipes)

12. 50× M solution (see Recipes)

13. 50 × 512 solution (see Recipes)

14. Complete autoinduction medium (AIM) (see Recipes)

15. 1 M Tris-HCl (see Recipes)

16. 4× SDS-PAGE sample buffer (see Recipes)

17. 10× buffer-H1 (see Recipes)

18. 26 mg/mL PMSF (see Recipes)

19. 200 mM benzamidine (see Recipes)

20. Lysozyme solution (see Recipes)

21. 2.5 mg/mL DNase I (see Recipes)

22. 10× Phosphate-Buffered Saline (PBS) (see Recipes)

23. PBS-Tween (PBS-T) (see Recipes)

24. Blocking solution (see Recipes)

25. 4 M NaCl (see Recipes)

26. 0.5 M TCEP (see Recipes)

27. 20% w/v Anzergent 3–12 (see Recipes)

28. Buffer-H2 (see Recipes)

29. 5 M imidazole (see Recipes)

30. Washing buffer (see Recipes)

31. Elution buffer (see Recipes)

**Recipes**

*Note: Solutions listed below can be stored at room temperature unless indicated otherwise.*

**1. Luria-Bertani broth (Miller) (LB)**

Prepare 500 mL of LB medium by adding the components listed below in an autoclavable bottle and dissolving them in 500 mL of ultra-pure (type 1) water. Sterilize by autoclaving at 121 °C for 30 min.

**Table:** 

Reagent	Final concentration	Quantity
Tryptone	10 g/L	5 g
NaCl	10 g/L	5 g
Yeast extract	5 g/L	2.5 g

**2. 1 M MgSO_4_**

Dissolve 24.6 g of MgSO_4_·7H_2_O in 100 mL of ultra-pure (type 1) water. Sterilize by autoclaving at 121 °C for 30 min.

**3. 1 M MgCl_2_**

Dissolve 20.3 g of MgCl_2_·6H_2_O in 100 mL of ultra-pure (type 1) water. Sterilize by autoclaving at 121 °C for 30 min.

**4. 50% glycerol**

Dissolve 50 mL of glycerol in 100 mL of ultra-pure (type 1) water. Sterilize by autoclaving at 121 °C for 30 min.

**5. Transforming and storage solution (TSS)**

Prepare 50 mL of TSS medium by dissolving the components listed below in 47.5 mL of LB medium. Adjust the pH of the solution to 6.5. Sterilize by filtration. Finally, add 2.5 mL of filter-sterilized DMSO in sterile conditions (final concentration 5% v/v). Store at 4 °C.

**Table:** 

Reagent	Final concentration	Quantity
PEG	10% w/v	5 g
1 M MgSO_4_	20 mM	1 mL
1 M MgCl_2_	20 mM	1 mL

**6. 100 mg/mL ampicillin**

Dissolve 5 g of ampicillin in 50 mL of ultra-pure (type 1) water. Sterilize by filtration. Store at 4 °C.

**7. 3 M KCl**

Dissolve 22.36 g of KCl in 100 mL of ultra-pure (type 1) water.

**8. 4 M NaCl**

Dissolve 23.38 g of NaCl in 100 mL of ultra-pure (type 1) water.

**9. 40% (w/v) glucose**

Dissolve 20 g of glucose in 50 mL of ultra-pure (type 1) water. Sterilize by filtration.

**10. Super optimal broth with catabolite repression (SOC)**

Prepare 100 mL of SOC medium by dissolving the components listed below in ultra-pure (type 1) water. Adjust the pH of the solution to 7.0. Sterilize by filtration. Aliquot in 50 mL and store at 4 °C.

**Table:** 

Reagent	Final concentration	Quantity
Tryptone	20 g/L	2 g
Yeast extract	5 g/L	0.5 g
4 M NaCl	10 mM	250 μL
3 M KCl	2.5 mM	83.33 μL
1 M MgCl_2_	10 mM	1 mL
1 M MgSO_4_	10 mM	1 mL
40% (w/v) glucose	20 mM	900.9 μL

**11. Terrific broth**

Prepare 960 mL of terrific broth medium by dissolving the components listed below in ultra-pure (type 1) water. Sterilize by autoclaving at 121 °C for 30 min.

**Table:** 

Reagent	Final concentration	Quantity
Tryptone	12 g/L	12 g
Yeast extract	24 g/L	24 g

**12. 50× M solution**

Prepare 1 L of solution by dissolving the components listed below in ultra-pure (type 1) water. Sterilize by autoclaving at 121 °C for 30 min. Filter the solution in sterile conditions to avoid crystal formation.

**Table:** 

Reagent	Final concentration	Quantity
Na_2_HPO_4_·7H_2_O	1.25 M	335.1 g
KH_2_PO_4_	1.25 M	170 g
NH_4_Cl	2.5 M	134 g
Na_2_SO_4_	0.25 M	35.5 g

**13. 50× 512 solution**

Prepare 1 L of solution by dissolving the components listed below in ultra-pure (type 1) water. Sterilize by filtration.

**Table:** 

Reagent	Final concentration	Quantity
Glycerol	25% w/v	250 g
Glucose	5% w/v	50 g
α-lactose monohydrate	10% w/v	105.2 g

**14. Complete autoinduction medium (AIM)**

Prepare 1 L of AIM by adding the components listed below to 960 mL of sterile terrific broth medium under sterile conditions.

**Table:** 

Reagent	Final concentration	Quantity
1 M MgSO_4_	2 mM	2 mL
1,000**×** trace metal mixture	0.2**×**	200 μL
50**×** 512 solution	1**×**	20 mL
50**×** M solution	1**×**	20 mL
100 mg/mL ampicillin	0.4 mg/mL	4 mL

**15. 1 M Tris-HCl**

Dissolve 12.1 g of Tris buffer in 70 mL ultra-pure (type 1) water. Adjust to the desired pH with HCl. Complete to 100 mL with ultra-pure (type 1) water. Sterilize by autoclaving at 121 °C for 30 min.

**16. 4× SDS-PAGE sample buffer**

Prepare 50 mL of solution by adding the components listed below and complete the final volume with ultra-pure (type 1) water. Aliquot 1 mL in 1.5 mL microcentrifuge tubes and store at -20 °C. This recipe is for preparing a reducing buffer. Replace the 2-mercaptoethanol with ultra-pure (type 1) water for a non-reducing buffer.

**Table:** 

Reagent	Final concentration	Quantity
1 M Tris pH 6.8	250 mM	12.5 mL
SDS	8% w/v	4 g
Glycerol	40% v/v	20 mL
Bromophenol blue sodium salt	0.4% w/v	200 mg
2-Mercaptoethanol	20% v/v	10 mL

**17. 10× buffer-H1**

Prepare 1 L of solution by dissolving the components listed below in ultra-pure (type 1) water. Adjust the pH of the solution to 8.0. To prepare 1× solution, dilute 10 times with ultra-pure (type 1) water and adjust the pH to 8.0 if necessary.

**Table:** 

Reagent	Final concentration	Quantity
Tris	500 mM	60.6 g
NaCl	1.5 M	87.7 g

**18. 26 mg/mL PMSF**

Dissolve 1.3 g of PMSF in 50 mL of isopropanol. Store at room temperature, protected from light.

**19. 200 mM benzamidine**

Dissolve 1.64 g of benzamidine hydrochloride hydrate in 50 mL of cold ultra-pure (type 1) water. Aliquot and store at -20 °C.

**20. Lysozyme solution**

Prepare 50 mL of solution by supplementing cold 1× buffer-H1 with the components listed below. Prepare just before use and keep at 4 °C.

**Table:** 

Reagent	Final concentration	Quantity
200 mM benzamidine	1 mM	250 μL
26 mg/mL PMSF	0.17 mg/mL	327 μL
2-Mercaptoethanol	2 mM	7 μL
Lysozyme	0.5 mg/mL	25 mg

**21. 2.5 mg/mL DNase I**

Prepare 20 mL of solution by dissolving the components listed below in cold, ultra-pure (type 1) water. Aliquot 1 mL in 1.5 mL microcentrifuge tubes and store at -20 °C.

**Table:** 

Reagent	Final concentration	Quantity
1 M Tris pH 7.5	20 mM	0.8 mL
1 M MgCl_2_	1 mM	40 μL
DNase I	2.5 mg/mL	100 mg
Glycerol	50% v/v	20 mL

**22. 10× phosphate-buffered saline (PBS)**

Prepare 1 L of solution by dissolving the components listed below in ultra-pure (type 1) water. Adjust the pH of the solution to 7.5. To prepare 1× solution, dilute 10 times with ultra-pure (type 1) water.

**Table:** 

Reagent	Final concentration	Quantity
Na_2_HPO_4_·7H_2_O	100 mM	26.8 g
NaCl	1.37 M	80 g
KCl	27 mM	2 g
KH_2_PO_4_	18 mM	2.4 g

**23. PBS-Tween (PBS-T)**

Add 0.5 mL of Tween 20 to 1 L of 1× PBS (0.05% Tween 20).

**24. Blocking solution**

Dissolve 2.5 g of low-fat dry milk in 50 mL of PBS-T.

**25. 4 M NaCl**

Dissolve 233.76 g of NaCl in 1 L of ultra-pure (type 1) water.

**26. 0.5 M TCEP**

Prepare 40 mL of solution by dissolving 5.733 g of TCEP-HCl in 25 mL of chilled ultra-pure (type 1) water. Bring the solution’s pH to 7.0 with a 10 M NaOH solution (around 7.5 mL). Complete the final volume to 40 mL, aliquot, and store at -20 °C.

**27. 20% w/v Anzergent 3–12**

Prepare 50 mL of solution by dissolving 10 g of Anzergent 3–12 in 50 mL of ultra-pure (type 1) water. Store at 4 °C.

**28. Buffer-H2**

Prepare 100 mL of solution by adding the components listed below in ultra-pure (type 1) water. Adjust the pH of the solution to 8.0. Since the 1× buffer-H1 contains 150 mM NaCl, adding 350 mM NaCl results in a final concentration of 500 mM NaCl.

**Table:** 

Reagent	Final concentration	Quantity
10× buffer-H1	1×	10 mL
4 M NaCl	350 mM (500 mM total)	8.75 mL
20% w/v Anzergent 3–12	0.4% w/v	2 mL

**29. 5 M imidazole**

Prepare 200 mL of solution by dissolving 68.08 g of imidazole in 100 mL of ultra-pure (type 1) water. Adjust pH to 8.0. Complete volume to 200 mL with ultra-pure (type 1) water.

**30. Washing buffer**

Prepare 100 mL of solution by adding the components listed below in ultra-pure (type 1) water. Adjust the pH of the solution to 8.0. Since the 1× buffer-H1 contains 150 mM NaCl, adding 350 mM NaCl results in a final concentration of 500 mM NaCl. Supplement with 2 mM TCEP (400 μL of 0.5 M TCEP) and 0.17 mg/mL PMSF (654 μL of 26 mg/mL PMSF) just before use.

**Table:** 

Reagent	Final concentration	Quantity
10× buffer-H1	1×	10 mL
4 M NaCl	350 mM (500 mM total)	8.75 mL
5 M imidazole	50 mM	1 mL
20% w/v Anzergent 3–12	0.4% w/v	2 mL

**31. Elution buffer**

Prepare 100 mL of solution by adding the components listed below in ultra-pure (type 1) water. Adjust the pH of the solution to 8.0. Since the 1× buffer-H1 contains 150 mM NaCl, adding 350 mM NaCl results in a final concentration of 500 mM NaCl. Supplement with 2 mM TCEP (400 μL of 0.5 M TCEP) just before use.

**Table:** 

Reagent	Final concentration	Quantity
10× buffer-H1	1×	10 mL
4 M NaCl	350 mM (500 mM total)	8.75 mL
5 M imidazole	500 mM	10 mL
20% w/v Anzergent 3–12	0.4% w/v	2 mL

**Laboratory supplies**

1. Pyrex^®^ round media storage bottles and reusable screw caps (Corning, catalog number: CLS1395)

2. Pyrex^®^ Vista^TM^ test tubes 25 × 150 mm (Corning, catalog number: 70800)

3. Foam plugs for test tubes and laboratory flasks (Chemglass Life Sciences, catalog number: CGE-1490)

4. Pyrex^®^ baffled shaker flasks (Corning, catalog number: 4444)

5. Sterile 15 mL and 50 mL conical polypropylene centrifuge tubes (Thermo Scientific, catalog number: 339650)

6. Sterile 1.5 mL microcentrifuge tubes (Corning, catalog number: MCT-150-C)

7. Falcon^®^ sterile polypropylene round-bottom tubes (Corning, catalog number: 352059)

8. PYREX^®^ 2800 mL Fernbach-style culture flask with baffles (Corning, catalog number: 4423-2XL)

9. Glass beads, acid-washed (Sigma-Aldrich, catalog number: G8772)

10. 0.6 mL microcentrifuge tubes (Corning, catalog number: MCT-060-C)

11. 1 L polycarbonate bottle assembly (Beckman Coulter, catalog number: C31600)

12. PVDF membrane (Bio-Rad, catalog number: 1620264)

13. His-Pur Ni-NTA resin (Thermo Scientific, catalog number: 88222)

14. Econo-Pac^®^ chromatography column (Bio-Rad, catalog number: 7321010)

15. Vivaspin^®^ Turbo 15 PES centrifugal concentrator (Sartorius, catalog number: VS15T32)

16. ENrich SEC 650 column (Bio-Rad, catalog number: 7801650)

17. Disposable polystyrene cuvettes (Bio-Rad, catalog number: 2239955)

## Equipment

1. Synergy UV R water purification system (Millipore, model: SYNSVR000)

2. Incubator shaker (Infors HT, model: Multitron, SM100116-HC)

3. Refrigerated centrifuge (Beckman Coulter, model: Avanti J-26 XPI)

4. JS-5.3 AllSpin swinging-bucket rotor and buckets (Beckman Coulter, catalog number: 368690)

5. Water bath (Fisher Scientific, model: 202S)

6. Spectrophotometer to measure OD_600_ (Implen, model: DiluPhotometer OD600)

7. Benchtop microcentrifuge (Fisher Scientific, model: accuSpin Micro 17R)

8. J-LITE JLA-8.1000 fixed-angle aluminum rotor (Beckman Coulter, catalog number: 363688)

9. Digital sonifier (Branson, model: SFX 250)

10. Ultracentrifuge (Beckman, model: Optima XL-80K)

11. Type 45 Ti fixed-angle titanium rotor (Beckman Coulter, catalog number: 339160)

12. Electrophoresis chamber (Bio-Rad, catalog number: 1658004)

13. Power supply (Bio-Rad, catalog number: 1645050)

14. Gel Doc EZ system (Bio-Rad, catalog number: 1708270)

15. Stain-free sample tray (Bio-Rad, catalog number: 1708274)

16. Trans-Blot^®^ Turbo^TM^ transfer system (Bio-Rad, catalog number: 1704150)

17. ChemiDoc MP imaging system (Bio-Rad, catalog number: 12003154)

18. NGC Quest 10 Plus chromatography system (Bio-Rad, catalog number: 7880003)

19. Spectrophotometer (Thermo Scientific, model: NanoDrop Lite)

20. Steam sterilizer autoclave (Market Forge, model: STM-E)

## Procedure

**A. Preparation of chemically competent cells**

*Note: We have observed that fresh competent cells produce higher yields of recombinant protein in* E. coli. *Therefore, we routinely prepare our own competent cells using the procedure by Chung and Miller [20].*

1. Starting from the frozen BL21-Gold(DE3) cells stock, transfer some bacteria using a pipette tip to grow a preculture in 5 mL of LB medium without antibiotics overnight at 37 °C and 250 rpm in a sterile Pyrex Vista^TM^ test tube. To ensure the medium is sterile, prepare a control tube with 5 mL of LB medium without cells and incubate it under the same conditions as the culture.

**Critical:** All materials and solutions should be sterile. Procedures should be performed under sterile conditions under a Bunsen burner flame. Sterility when working with bacteria is critical to obtain final high protein yields.

2. The next day, dilute the saturated culture 1:100 in LB with no antibiotics. For instance, add 250 μL of saturated culture in 25 mL of LB in a 125 mL baffled shaker flask or 500 μL of saturated culture in 50 mL of LB in a 250 mL baffled shaker flask.

*Note: The optical density at 600 nm (OD_600_) of the saturated culture is between 4.5 and 5.0.*

3. Grow cells at 37 °C and 250 rpm until OD_600 _= 0.5 (around 2 h) by adding 1 mL of the culture in a cuvette and measuring its optical density at 600 nm. In the meantime, chill tubes to receive the culture on ice. Turn on and chill the centrifuge to 4 °C so it is cold to harvest cells later.

**Critical:** Do not overgrow cells.

4. Transfer the culture flask to ice and incubate for 30 min.

**Critical:** All subsequent steps are carried out at 4 °C. Keeping the cells cold is critical to obtain high transformation efficiencies.

5. Transfer cells to a sterile chilled conical tube and centrifuge at 1,200× *g* for 10 min at 4 °C.

6. Gently resuspend the cells in 1/10 of the initial harvested volume with chilled TSS (for instance, for 15 mL of harvested culture, resuspend in 1.5 mL of TSS).

7. Aliquot 120 μL of cells in sterile chilled 1.5 mL centrifuge tubes, flash-freeze, and store at -80 °C. These aliquots can be used for three months if kept at -80 °C.

*Note: As reported by Chung and Miller [20], the typical transformation efficiency of these cells is ~10^7^ CFU/μg DNA.*

**B. Transformation**

1. Add 1 μL of the pHis-EK-hH_v_1.pET15-b plasmid (100–200 ng of DNA) to a chilled sterile polypropylene round-bottom tube on ice.

2. Thaw one aliquot of BL21-Gold(DE3) competent cells on ice.

3. Add 100 μL of competent cells to the bottom of the tube where the DNA was placed. Gently mix three times.

4. Incubate the cells with DNA for 5 min on ice.

5. Apply thermal shock by placing the tube in a water bath at 42 °C for 45 s.

6. Incubate cells on ice for 2 min at 4 °C.

7. Add 1 mL of SOC at room temperature to the tube.

8. Grow cells for 1 h at 37 °C and 250 rpm.

9. Inoculate cells in a 125 mL baffled flask containing 25 mL of LB media supplemented with 0.2% (w/v) glucose [125 μL of 40% (w/v) glucose] and 0.4 mg/mL ampicillin (100 μL of 100 mg/mL ampicillin). Grow overnight at 37 °C and 250 rpm.

**C. Protein expression**

Protein expression is accomplished by using the protocol for autoinduction by Studier with minor modifications [21]. Before starting the culture for expression, 960 mL of sterile terrific broth must be prepared in a 2.8 L baffled flask the previous day.

1. Prepare complete AIM (see Recipes) on the day of the experiment.

2. Dilute the saturated preculture 1:100 in the complete AIM (10 mL in 1 L).

3. Grow the culture at 37 °C and 250 rpm until the OD_600 _= 1.5 (around 2–3 h).

3. Transfer the culture to a shaker previously set to 20 °C.

4. Grow overnight at 20 °C and 250 rpm.

**D. Cell harvesting and lysis**

1. Determine the OD_600_ and collect samples for western blot.

a. Measure the OD_600_ of a 1:20 dilution of the culture (50 μL in 950 μL of LB media). Recover the OD_600_ of the culture by multiplying the measured OD_600_ by 20.

b. Take an aliquot (V) of the culture in a 1.5 mL microcentrifuge tube using the following equation: V (μL) = 800/OD_600_.

c. Centrifuge the sample in a benchtop microcentrifuge at 3,500× *g* for 5 min at 4 °C.

d. Discard the supernatant and lyse bacteria by adding 250 μL of 1× SDS-PAGE sample buffer.

**Caution:** Make sure to dissolve the cell pellet by pipetting it with the sample buffer. Be careful not to accidentally lose the pellet in the pipette tip during this procedure.

e. Add 300 μL of glass beads to the extract (measured with a 0.6 mL microcentrifuge tube). Vortex the sample for 2 s, 20 times.

f. Store samples at -20 °C.

2. Add 500 μL of 10% v/v antifoam 204 to the culture and mix.

3. Transfer the culture to a 1 L polycarbonate bottle to harvest cells at 4,000× *g* for 30 min at 4 °C.

4. Discard the supernatant and collect the cell pellet in a previously weighed 50 mL conical polypropylene centrifuge tube. Take note of the cell pellet mass (should be around 20 g).

5. Resuspend the pellet in a final volume of 40 mL with cold lysozyme solution and incubate the cell suspension for 30 min at 4 °C with gentle rotation. Store at -80 °C.

6. Thaw the cell extracts in a water bath at room temperature.

7. Supplement the thawed extract with 0.17 mg/mL PMSF, 1 mM benzamidine, 5 mM MgCl_2_, 2 mM 2-mercaptoethanol, and 12.5 μg/mL DNase I.

8. Incubate the extract for 1 h at 4 °C with gentle rotation.

9. Dilute the extract with cold 1× buffer-H1 supplemented with 0.17 mg/mL PMSF, 1 mM benzamidine, and 2 mM 2-mercaptoethanol to a final concentration of 0.2 g of cell pellet per milliliter.

10. Sonicate the extract in *Time Mode* for a total ON time of 6 min and 60% amplitude in a water-ice bath, with 7-s ON and 15-s OFF cycles.

**Critical:** Keep the temperature low (close to 4 °C). Use a glass container and the ice-water bath to optimize heat transfer. Also, set the sonifier tip deep enough and close to the base to avoid the formation of bubbles.

11. Centrifuge the sonicated extract at 100,000× *g* (30,000 rpm in a Type 45 Ti rotor) for 1 h at 4 °C.

12. Resuspend the membrane pellet with the help of a brush in cold 1× buffer-H1 supplemented with 0.17 mg/mL PMSF and 1 mM benzamidine. Measure the final volume of the membrane extract and take note of the concentration (mass of cell pellet per milliliter). The concentration should be between 0.5 and 0.4 g/mL.

13. Aliquot the membrane extract and store it at -80 °C.

**E. Gel electrophoresis and western blot**

*Note: Before proceeding with protein purification, we strongly recommend checking that the protein was expressed by performing a western blot against the His-tag.*

**Figure 1.**
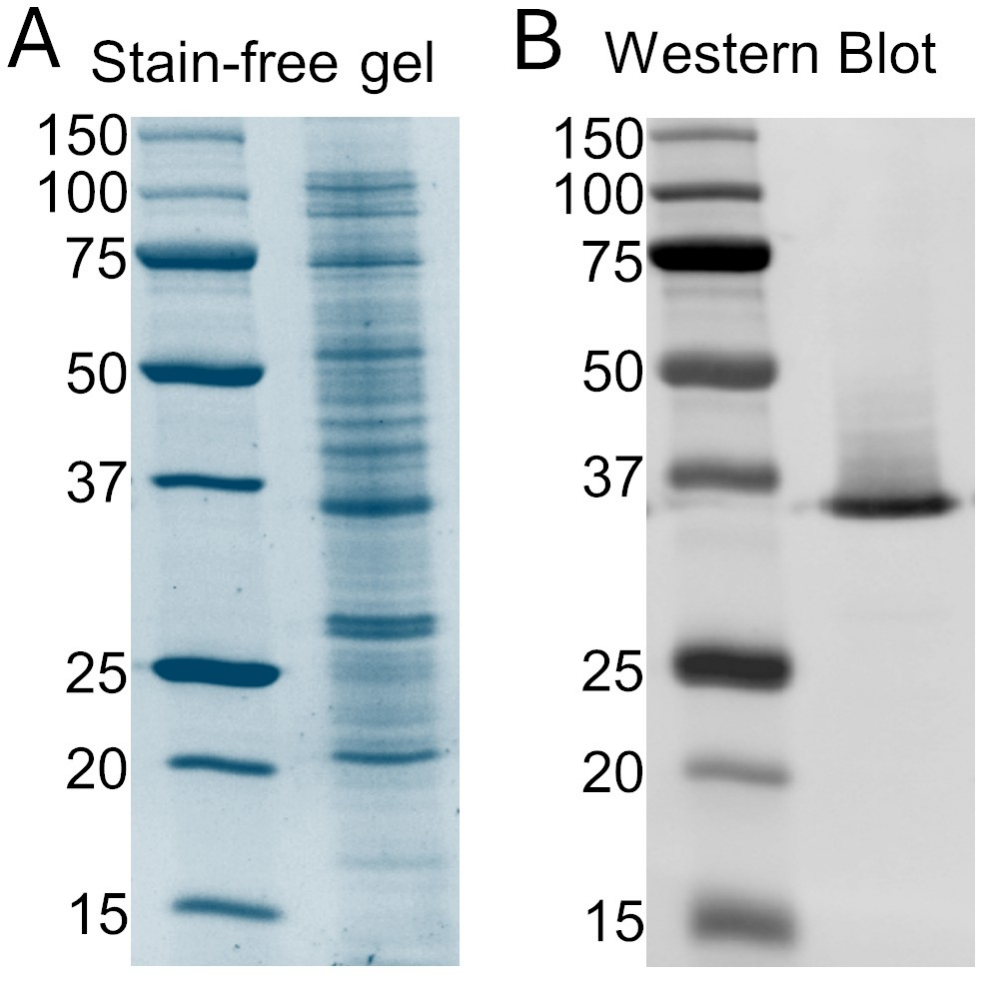
Expression of EK-hH_v_1. (A) Stain-free gel of cellular extract. The left lane is the protein ladder, and the right lane corresponds to the cellular extract sample. (B) Western blot against the poly-His tag of the cellular extract. The left lane is the protein ladder, and the right lane corresponds to the cellular extract sample. hH_v_1: human voltage-gated proton channel.

1. Perform an SDS-PAGE electrophoresis of the samples prepared before harvesting cells (step D1).

a. Open and install a 12% Mini-PROTEAN^®^ Stain-Free™ protein gel according to the manufacturer’s instructions.

b. Use 6 μL of a 1:1 mixture of unstained standards and dual color standards as the protein ladder.

c. Load 15–20 μL of sample.

d. Run electrophoresis at 120 V for 70 min.

e. Disassemble the gel, activate, and image the stain-free gel (Figure 1A).

f. Place the gel in 1× transfer buffer and incubate with gentle agitation for at least 5 min.

2. Activate a PVDF membrane for the western blot:

a. Cover the membrane with ethanol (10–15 mL).

b. Discard the ethanol and add enough 1× transfer buffer to cover the membrane completely.

c. Incubate with gentle agitation at room temperature for at least 5 min.

**Caution:** Manipulate the PVDF membrane using clean gloves and forceps.

3. Transfer the proteins from the gel to the membrane following the instructions of the Trans- Blot^®^ Turbo^TM^ transfer system. Use a constant 2.5 A for 4 min.

4. Incubate the membrane in 50 mL of fresh blocking solution for 1 h at room temperature with gentle agitation.

5. Remove the milk and add the primary antibody (50 μL of mouse α-penta-His antibody in 15 mL of PBS-T). Incubate overnight at 4 °C with gentle agitation.

6. Next day, remove the primary antibody solution and wash the membrane with PBS-T.

a. Wash the membrane by adding, manually agitating, and discarding PBS-T three times.

b. Add PBS-T and incubate at room temperature for 5 min with gentle agitation.

c. Repeat the two previous steps three times.

7. Discard the last wash and add the secondary antibody (2 μL of StarBright Blue 700 goat α-mouse IgG in 15 mL of PBS-T). Incubate for 1 h at room temperature with gentle agitation.

8. Remove the secondary antibody solution and wash the membrane with PBS-T.

a. Wash the membrane by adding, manually agitating, and discarding PBS-T three times.

b. Add PBS-T and incubate at room temperature for 5 min with gentle agitation.

c. Repeat the two previous steps three times.

9. Image the membrane with a ChemiDoc MP imaging system following the equipment instructions for capturing the fluorescent signal of the secondary antibody (Figure 1B).

**F. Solubilization**

1. Thaw the membrane extract in a water bath at room temperature. To solubilize the protein, first determine the final volume needed to have the extract at a final concentration of 0.16 g/mL. Using this final volume, add 500 mM NaCl, 12.5 μg/mL DNase, 5 mM MgCl_2_, 2 mM TCEP, 0.17 mg/mL PMSF, 1 mM benzamidine, and 1.5% w/v Anz3–12. Complete the volume with 1× buffer-H1. As an example, the volumes added from the stock solutions for solubilizing 10 mL of a membrane extract at an initial concentration of 0.32 g/mL are shown in Table 1.

**Table 1.** Solubilization example. For the solubilization of 25 mL of membrane extract at 0.32 g/mL, the final volume should be 50 mL. Below are the volumes added from the stock solutions to solubilize this extract.

Stock solution	Final concentration	Volume
0.32 g/mL membrane extract	0.16 g/mL	25 mL
200 mM benzamidine	1 mM	250 μL
26 mg/mL PMSF	0.17 mg/mL	327 μL
1 M MgCl_2_	5 mM	250 μL
2.5 mg/mL DNase I	12.5 μg/mL	250 μL
4 M NaCl	500 mM	6.25 mL
0.5 M TCEP	2 mM	200 μL
20% w/v Anzergent 3–12	1.5% w/v	3.75 mL
1× buffer-H1		13.7 mL

2. Incubate the solution for 1 h at room temperature with gentle rotation.

3. Centrifuge the solution at 100,000× *g* (30,000 rpm in a Type 45 Ti rotor) for 1 h at 4 °C.

**G. Purification**

1. Pack and equilibrate a Ni-NTA gravity-flow column:

a. Pour 10 mL of Ni-NTA resin per liter of culture (20 mL of a 50% slurry) into a gravity-flow column and drain the storage buffer.

b. Wash the column with at least 10 column volumes (CVs) of ultra-pure (Type 1) water.

c. Equilibrate the column with 6 CVs of buffer-H2.

2. Binding in batch:

a. Filter the supernatant of the centrifuged solubilized membrane extract with a 0.45 μm membrane filter and supplement with fresh 1 mM benzamidine.

b. Mix the supernatant with the equilibrated resin and incubate for 1 h at room temperature with gentle rotation.

c. Collect the resin in a gravity-flow column.

3. Wash the resin with 10 CVs of washing buffer.

4. Elution of the purified EK-hH_v_1:

a. Add one CV of elution buffer. Collect the eluate. Usually, this eluate contains only small amounts of EK-hH_v_1 protein.

b. Stop the flow of the column and add one CV of elution buffer. Incubate the column for 10 min at room temperature.

c. Collect the eluate.

d. Repeat the two previous steps 8 times to recover a total of 10 CV of eluate containing purified EK-hH_v_1.

**Figure 2.**
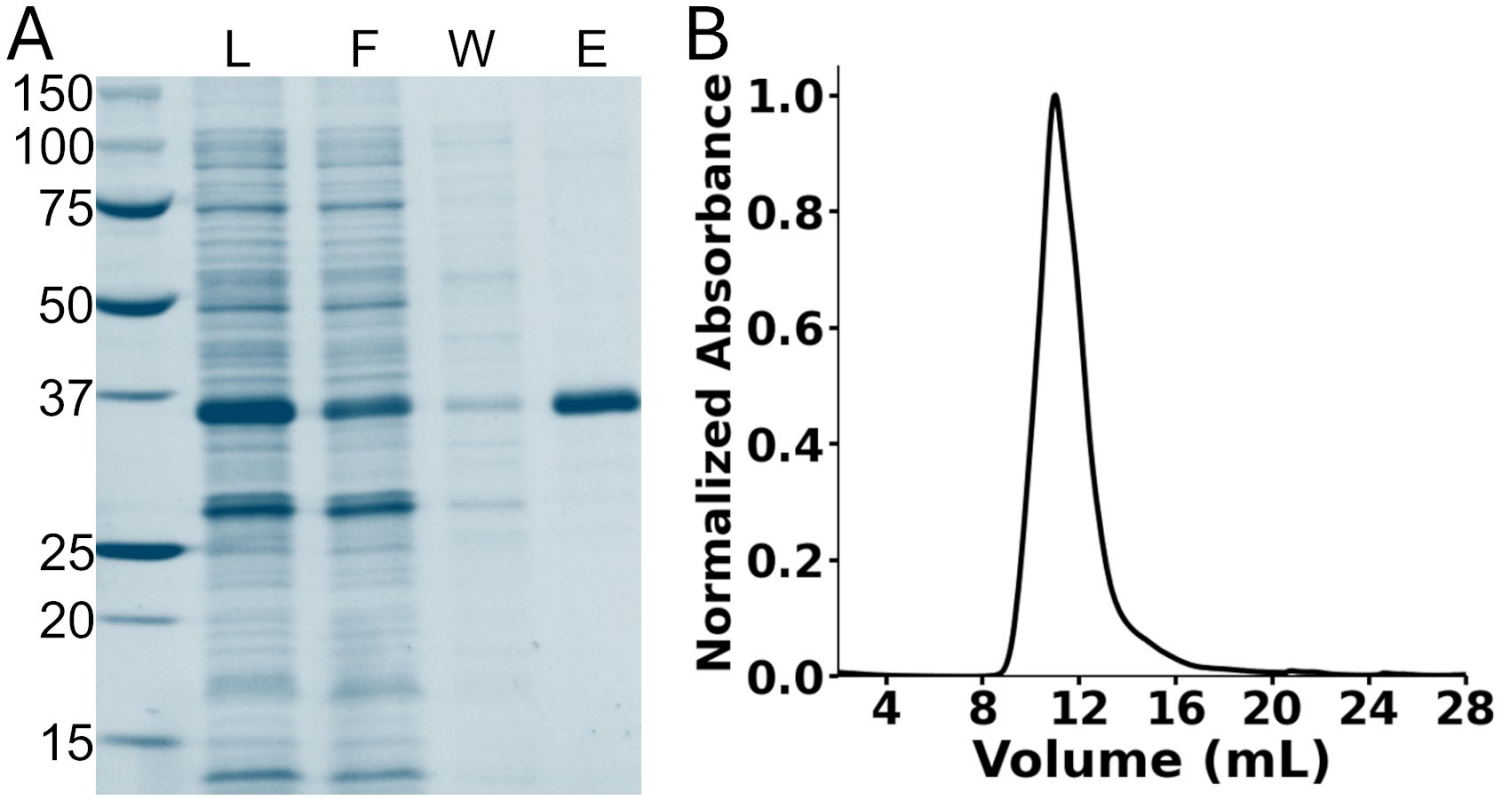
Purification of EK-hH_v_1. (A) Stain-free gel of the nickel immobilized metal affinity chromatography purification steps. The first lane corresponds to the protein ladder. L: Load; F: flowthrough; W: washing; E: elution. (B) Chromatogram of the size-exclusion chromatography of the purified EK-hH_v_1. hH_v_1: human voltage-gated proton channel.

5. Concentrate the desired amount of protein with a 50 kDa cutoff centrifugal filter by centrifuging at 1,200× *g* and 4 °C to at least 1 mL. The duration of this step will depend on the amount of protein to be concentrated.

6. Inject the sample into an ENrich SEC 650 column, which has been previously equilibrated with 1.5 CVs of buffer-H2 at room temperature. The EK-hH_v_1 protein will elute at around 11.0 mL (Figure 2).

7. Determine the EK-hH_v_1 protein concentration (C) by measuring the absorbance at 280 nm (Abs280) and using the equation C (mg/mL) = 1.20 (Abs280).

8. Concentrate the EK-hH_v_1 with a 50 kDa cutoff centrifugal filter to 5 mg/mL, aliquot, flash-freeze, and store at -80 °C.

## Validation of protocol

This protocol has been used and validated in the following research article:

- Carmona et al. [19]. A novel method for expressing and purifying large quantities of functional and stable human voltage‐gated proton channel (hH_v_1). *Protein Science* (Figures 1–5).

## General notes and troubleshooting

**General notes**

1. Purification can be performed with 150 mM NaCl instead of 500 mM NaCl, but the protein yield will decrease from 70 mg/L to 30 mg/L [19].

**Troubleshooting**

Problem 1: Low protein expression.

Possible causes: Low efficiency of transformation of competent cells. Contamination of sterile media.

Solutions: Prepare new competent cells. Replace the media.

Problem 2: Eluted EK-hH_v_1 is contaminated.

Possible cause: The washing buffer is draining too fast from the column.

Solution: Add the washing buffer in two CV steps, incubating for 5 min between steps.

## Supplementary information

The following supporting information can be downloaded here:

1. Dataset S1. Information and sequence of the pHis-EK-hH_v_1.pET15-b
